# A Fatigue Damage Model for Life Prediction of Injection-Molded Short Glass Fiber-Reinforced Thermoplastic Composites

**DOI:** 10.3390/polym13142250

**Published:** 2021-07-09

**Authors:** Mohammad Amjadi, Ali Fatemi

**Affiliations:** Mechanical Engineering Department, University of Memphis, Memphis, TN 38152, USA; mamjadi@outlook.com

**Keywords:** thermoplastics, short-fiber composites, fatigue, damage mechanics, injection molding

## Abstract

Short glass fiber-reinforced (SGFR) thermoplastics are used in many industries manufactured by injection molding which is the most common technique for polymeric parts production. Glass fibers are commonly used as the reinforced material with thermoplastics and injection molding. In this paper, a critical plane-based fatigue damage model is proposed for tension–tension or tension–compression fatigue life prediction of SGFR thermoplastics considering fiber orientation and mean stress effects. Temperature and frequency effects were also included by applying the proposed damage model into a general fatigue model. Model predictions are presented and discussed by comparing with the experimental data from the literature.

## 1. Introduction

Short glass fiber-reinforced (SGFR) thermoplastics have been gaining more attention in many industries during recent decades. The motivation for manufacturing reinforced polymeric composites is to get the most advantages of each polymer matrix and reinforcement materials. Polymer properties can be improved by adding fibers. Fiber materials can be inorganic (glass or metals), synthetic (carbon), natural (wood or bamboo), or other highly oriented polymers. The orientation of the fibers relative to one another, fiber concentration with respect to applied stresses, and fiber distribution have significant effects on mechanical properties of fiber-reinforced composites.

Short glass fiber-reinforced polymer matrix composites can be made of thermosets or thermoplastics. Thermoplastics mechanical properties are usually enhanced with short, discontinuous fibers, while thermosets are filled with long, continuous fibers. Although continuous fiber composites have much better stiffness and strength compared to short fiber composites, they are more difficult to process and much more costly. In addition, thermoplastic matrix composite parts can be joined by heating, are recyclable, and have better toughness and impact resistance than thermosets.

Modulus, strength, creep performance and the service temperature of neat polymers increase by adding fibers. Therefore, it is possible to use polymeric composites in structural and load bearing applications, for example in the automotive industry to reduce automobile weight, typically with injection molding manufacturing technique.

During injection molding, the induced shear to the melted polymer might cause aligning of the molecular chains and this mechanical deformation creates a structure regularity along the flow direction. In addition, as melt flow cools down, the orientation of molecules in flow direction is frozen. Therefore, tensile strength in the flow direction may be higher than cross-flow direction. Mold temperature, part thickness and flow thickness can affect the orientation of polymer molecules in injection molding.

Short fiber polymeric composites injection-molded to complex geometries at a very high production rate are very attractive to the automotive industry. Replacing steel with polymers and polymeric composites in the automotive industry is expected to increase the demand for injection-molded polymers over the next decade [1].

Polymeric composites structural parts typically undergo complex and cyclic mechanical loadings along with environmental condition variations such as temperature and humidity. Therefore, fatigue design with polymeric composites is a serious challenge.

The aim of this study was to develop and propose a critical plane damage model for tension–tension or tension–compression fatigue life estimation of short glass fiber-reinforced thermoplastics. Mean stress, fiber orientation, temperature, and frequency effects are included in this study. Uniaxial fatigue data sets from literature for injection-molded SGFR thermoplastics with oriented fibers were used as illustrative examples in this study in order to evaluate the applicability of the proposed damage model and accounting for the aforementioned effects.

## 2. Damage Mechanisms in Short Reinforced Fiber Composites

Deformation and damage mechanisms in neat thermoplastics under multiaxial cyclic loading were investigated and discussed in [2]. It was observed that shear failure is the dominant damage mode and crack initiation mechanism for studied neat thermoplastics including High-Density Polyethylene (HDPE), Polypropylene (PP), and PA66 (Polyamide 66) under cyclic loading [2]. However, crack initiation mechanisms are different for short glass fiber-reinforced thermoplastics in presence of glass fibers in the polymer matrix. Defects and flaws formed during production or service loading in the fiber, matrix, and/or the fiber-matrix interface are the critical locations for damage initiation and subsequent growth to fracture. Parameters such as fiber content, orientation, as well as aspect ratio in addition to interfacial bonding (adhesion), and matrix behavior influence the behavior of short fiber-reinforced thermoplastics.

The fiber volume fraction is a manufacturing parameter for injection-molded SGFR thermoplastics and the higher the volume fraction, the better the fatigue performance as is the case for monotonic loading. However, the slope of S–N curves was observed to be independent of fiber volume content for PA66 [3]. Janzen and Ehrenstein [4] investigated the fatigue behavior of glass fiber-reinforced polybutylene terephthalate (PBT) with fiber volume fraction varying from 15 to 44%. It was observed that fatigue strengths in low cycle fatigue region significantly depended on the fiber content and additional fibers resulted in higher strength. On the other hand, in high cycle fatigue region, the fiber content did not affect fatigue life.

Fiber orientation (FO) can be affected by thicknesses or injection gate locations. The skin/core fiber orientation ratio may vary as the part thickness increases. As the fiber orientation increases, the fatigue performance improves along the preferred fiber orientation direction. Fatigue performance in the transverse direction (perpendicular to the fiber orientation direction) is significantly lower than in the longitudinal direction [5,6].

Strong coupling or adhesion between fiber and matrix results in higher strength. Local debonding with formation of nanovoids are also necessary to activate matrix yielding. For SGFR thermoplastics with strong fiber-matrix bonding, damage occurs in the form of matrix-cracking at a certain distance from the interface rather than fiber-matrix debonding at the interface [3]. Failure mechanisms of SGFR thermoplastics may include many modes such as fiber breakage, fiber debonding and pull-out, shear crack formation along fibers, craze development and coalescence, matrix fracture, and crack branching [7].

**Figure 1:**
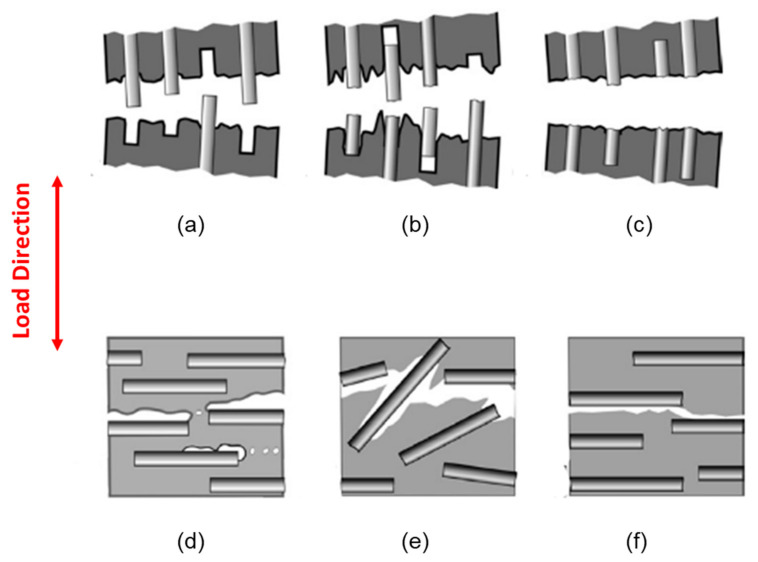
Deformation mechanisms for (**a**) poor, (**b**) partially good, and (**c**) good adhesion between fiber and matrix where the fibers are oriented along the loading direction. Deformation mechanisms for (**d**) poor, (**e**) partially good, and (**f**) good adhesion between fiber and matrix where the fibers are oriented perpendicular to the loading direction [8].

As shown in Figure 1a for fibers oriented along the applied load, debonding at the fiber-matrix interface followed by fiber pull out occurs for poor adhesion between fiber and matrix (Figure 1a). However, fiber gliding and fiber breakdown occur for a partial interfacial adhesion (Figure 1b) and a strong interfacial adhesion (Figure 1c), respectively, when fibers are oriented along the applied load [8].

When the fibers are oriented perpendicular to the applied load direction, microvoid formation can occur at the fiber interface if the interfacial adhesion is poor, as shown in Figure 1d. Fibers tilted at a certain angle can also separate from the matrix if the interfacial adhesion is not very good (Figure 1e). Cracks grow along the interface with the fibers or inside the matrix following a smooth fracture surface, as shown in Figure 1f [8].

Klimkeit et al. [9] showed that fiber ends are stress concentration locations where cracks can nucleate. Moisture absorption is a factor that can reduce fiber-matrix bonding and facilitate fiber debonding. It significantly influences the mechanical properties of the SGFR Polyamides due to the water molecules that can interact with the amide groups in the amorphous phase of the polyamide matrix. As a result, chain bond strength reduces and the mobility of the chains of the polyamide increases [10]. Under dry condition the damage mechanisms for PA66-35GF (Polyamide 66 + 35% Glass Fiber) were matrix cracking, fiber-matrix interface failure, and fiber pull-out under dry condition [11,12]. Fiber pull-out is dominant as compared to fiber failure due to buckling where fiber failure was found to be more relevant for higher fiber volume fraction (>50 wt.%) [3]. It has been also observed that the interface of fibers acts as a critical location when the other surrounding fibers are under stress [13]. On the other hand, another observation suggested that damage mechanisms are the same in neat and short glass fiber-reinforced polyamide starting in the matrix [13].

## 3. Studied Materials

Uniaxial fatigue data of short glass fiber thermoplastics from the literature were used in this paper and a critical plane-based damage parameter is proposed to account for fiber orientation and mean stress effects. Much of the data are for Polyamides (PA) or nylons. They are one of the most commonly used thermoplastics, as unreinforced neat or reinforced composite materials with applications in many industries such as automotive and electronics [14]. The other materials considered are PBT-PET, PA6, PP, and PBT with 30–35% glass fiber.

The common mechanical and physical properties of the considered materials are reported in Table 1.

The glass fiber content for all considered materials are 30% or 35% with fiber diameter of 10 μm. The fiber aspect ratio (fiber length/fiber diameter) range for all the considered SFGR thermoplastics in this paper is 23–26. Glass transition and melt temperatures as well as ultimate tensile strength and elastic modulus for each material at different temperatures are also included in Table 1. Uniaxial fatigue test conditions for short glass fiber-reinforced PBT, PBT-PET, PP, PA66, and PA6 thermoplastics with different fiber orientations at different temperatures and *R* ratios are listed in Table 2.

**Figure 2:**
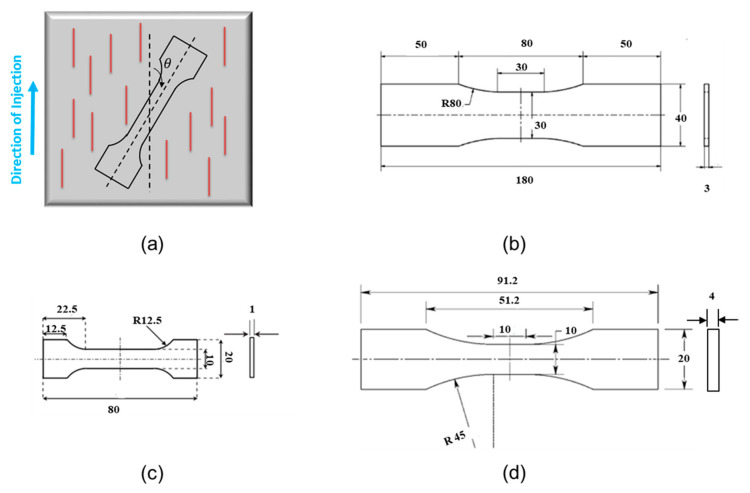
(**a**) Cutting the specimens with respect to injection direction (**b**) Specimen geometry used for uniaxial fatigue testing for PBT + PET-30GF from [9]. (**c**) Specimen geometry used for uniaxial fatigue testing for PA66-35GF from [18]. (**d**) Specimen geometry used for uniaxial fatigue testing for PP-30GF, PA66-30GF (Polyamide 66 + 30% Glass Fiber), PBT-35GF, and PA6-35GF from [5,6,15,16].

Fiber orientation angle is defined in Figure 2a. The actual experimental data used in this study from uniaxial fatigue tests of PA66, PA6, PP, and PBT under dry conditions can be found in [5,16].

Uniaxial tension–tension or tension–compression fatigue testing are typically performed using plate or flat specimen geometries (Figure 2). Data with thermal failure due to high temperature rise from self-heating in fatigue tests are not included in this paper.

## 4. Proposed Critical Plane Damage Model

Fatigue failure of polymers begins with the initiation of a crack in a surface craze, at a surface defect, or at a stress concentration point [19,20]. In general, under cyclic loading, cracks initiate with typically a preferred orientation, depending on microstructural characteristics and multiaxial stress state in the material. The damage mechanism is a key consideration in order to develop a proper predictive fatigue model.

The FS critical plane damage parameter has been used for multiaxial fatigue life prediction of metallic materials for three decades [21]. The original from of FS damage parameter can be expressed as:

(1) $$D=\frac{\Delta \gamma_{max}}{2}\left(1+k\frac{\sigma_{n,max}}{S_{y}}\right),$$

where $\Delta \gamma_{max}$ is maximum shear strain range, $\sigma_{n,max}$ is the maximum normal stress acting on the maximum shear strain plane over the cycle, and $S_{y}$ is the yield strength. Mean normal stress is accounted for since the maximum normal stress is the addition of mean stress, $\sigma_{n,m}$, and alternating stress, $\sigma_{n,a}$, so that $\sigma_{n,max}=\sigma_{n,m}+\sigma_{n,a}$. In this equation, k is a material constant found by superimposing pure axial and pure torsion data together. Gates and Fatemi [22] suggested to replace yield strength in Equation (1) with GΔγ to better account for the mean stress effect.

A stress version of this damage parameter was recently shown to satisfactory correlate multiaxial fatigue data for a variety of neat thermoplastics as well as reinforced PA66 with randomly short glass fiber orientation [2]. The stress version of this parameter in terms of axial fatigue properties can be written as Equation (2):

(2) $$D=\tau_{a}\left(1+k\frac{\sigma_{n,max}}{\tau_{a}}\right)\left(\frac{2}{1+k}\right)=\sigma_{f}^{\prime }\;{\left(2N_{f}\right)}^{b}$$

where $\sigma_{f}^{\prime },\;\mathrm{and}\;b$ are fatigue strength coefficient and fatigue strength exponent, respectively, and $\tau_{a}$ is the shear stress amplitude on the critical damage plane.

However, in the case of the thermoplastics with oriented short glass fibers, a modification to the critical plane damage parameter is needed. Based on the discussed damage mechanisms in injection-molded SGFR thermoplastics, matrix cracking at the fiber ends, fiber pull-out and debonding between fiber and matrix are the main failure modes. Therefore, the critical plane approach for neat thermoplastics is modified for application to uniaxial fatigue behavior of SGFR thermoplastics, as follows:

(3) $$D\left(\varphi \right)=\tau_{a}\cos\varphi \;\left[1+k\left(\frac{\;\sigma_{n,max}\;\sin\varphi }{\tau_{a}\cos\varphi \;}+\frac{\;\sigma_{n,max}\;\cos\varphi }{\tau_{a}\cos\varphi \;}+\frac{f\left(\;\sigma_{n,max},\tau_{a},\;\;\theta \right)}{\tau_{a}\cos\varphi \;})\right)\right],$$

(4) $$D_{C}\;\left(\frac{2}{1+k}\right)=\sigma_{f}^{\prime }\;{\left(2N_{f}\right)}^{b},$$

where θ is the orientation angle of aligned fibers with respect to the specimen axis and $f\left(\;\sigma_{n,max},\tau_{a},\;\theta \right)$ is a function in order to account for fiber orientation effect. Equation (4) can be used as the reference S-N line.

**Figure 3:**
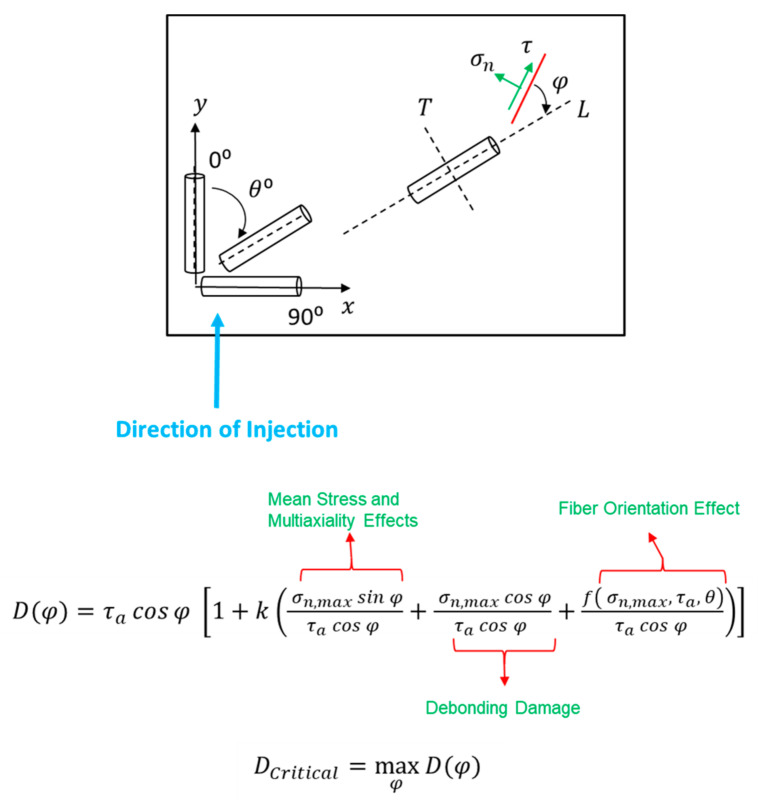
Proposed critical plane damage parameter for fatigue life prediction of oriented reinforced glass fiber thermoplastic composites.

As shown in Figure 3, the damage parameter is expressed as a function of φ which is the damage plane angle. Angle φ represents the projection of the normal and shear stresses along the fiber. In Equation (3), mean stress and multiaxiality effects are accounted for through the first term in parenthesis, similar to the original FS damage parameter (Equation (1)). Debonding between fiber and matrix is accounted for through the second term in parenthesis in Equation (3) where debonding damage is assumed to be dependent on the stress perpendicular to the fiber orientation.

Fiber orientation effect is also considered in this model. Similar to stress concentration at both crack ends in an infinite plate containing a crack, the fiber in the polymer matrix could be thought of as a small crack with regards to stress concentration effect at the fiber ends. Therefore, the third term as $f\left(\;\sigma_{n,max},\tau_{a},\;\theta \right)$ in parenthesis in Equation (3) is added. This function depends on the stress state and fiber orientation. For uniaxial fatigue behavior of materials with different fiber orientations, $f\left(\;\sigma_{n,max},\;\theta \right)$ in Equation (3) is expressed as:

(5) $$f\left(\;\sigma_{n,max},\;\theta \right)=\sigma_{n,max}\;sin^{2}\theta ,$$

where the term $sin^{2}\theta$ in Equation (5) is inspired from the stress concentration factor (Mode I) of an inclined crack under axial loading. Therefore, the stress concentration at fiber ends is higher in the transverse direction (θ=90°) as compared to the longitudinal direction (θ=0). The terms in parenthesis in Equation (3) are normalized by the shear stress amplitude on the critical plane because shear stress plays the key a role in the described damage mechanisms.

Under uniaxial loading condition and having the fiber orientation angle, the damage parameter $D\left(\varphi \right)$ is maximized over different planes. The maximum value of $D\left(\varphi \right)$ and φ are the critical damage value and critical plane for crack initiation. Similar to the damage parameter for neat thermoplastics (Equation (2)), *k* can be estimated by superimposing pure axial and pure torsion data together. Values of *k* for each material at different temperatures are reported in Table 3. Based on Table 3, the *k* values are similar and equal to either 0.1 or 0.2 for all materials and temperatures.

The proposed critical plane damage parameter in Equation (3) does not explicitly consider fiber length or fiber content. However, the reference condition (fully-reversed axial fatigue data) inherently accounts for fiber length or aspect ratio and content in the matrix for each material. Also, the fiber content does not affect the slope of the S-N curves for axial condition [23].

## 5. Uniaxial Fatigue Data Correlations

In order to evaluate the applicability of the proposed damage parameter, fatigue data under uniaxial loading condition were considered. The proposed damage parameter (Equation (3)) was used for uniaxial fatigue data correlations of SGFR thermoplastics under different *R* ratios and fiber orientations at different temperatures.

**Figure 4:**
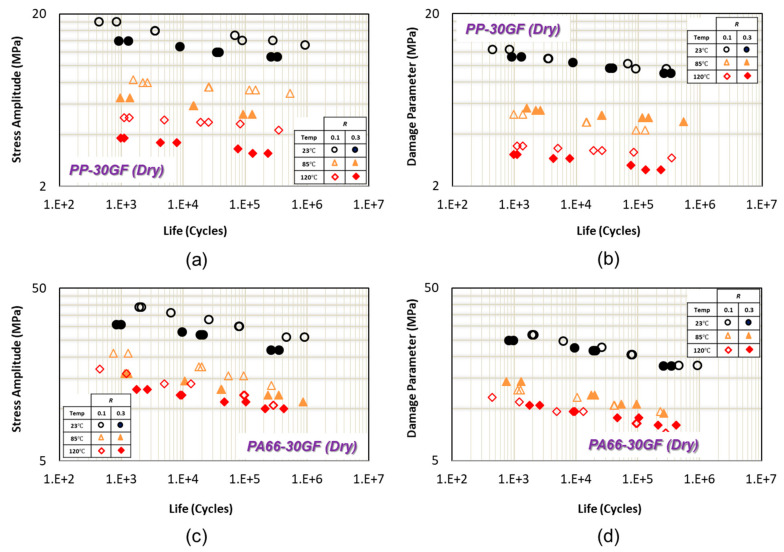
(**a**) Uniaxial fatigue data of PP-30GF in transverse direction from [15]. (**b**) Uniaxial fatigue data correlations of PP-30GF using the proposed damage parameter in transverse direction. (**c**) Uniaxial fatigue data of PA66-30GF in transverse direction from [15]. (**d**) Uniaxial fatigue data correlations of PA66-30GF using the proposed damage parameter in transverse direction.

In Figure 4a,c, uniaxial fatigue data for PP-30GF from and PA66-30GF from [15] are shown as the normal stress amplitude versus fatigue life in transverse (perpendicular to injection) direction at 23 °C, 85 °C, and 125 °C. As can be seen from Figure 4a,c, mean stress (i.e., at different *R* ratios) has a significant effect on fatigue life at each temperature.

Using the proposed damage parameter as Equation (3) with θ=90°, the uniaxial fatigue data at each temperature correlate well and get closer at two different R ratios (R = 0.1 and *R* = 0.3), as shown in Figure 4b,d for PP-30GF and PA66-30GF [15,16], respectively. Damage parameter (Equation (3)) is a combination of the maximum normal stress and its effect in terms of matrix/fiber bonding damage in addition to shear stress amplitude on critical plane. Therefore, the damage parameter (Equation (3)) can account for the mean stress or R ratio effect on fatigue life at a given temperature when fibers are oriented perpendicular to the axial loading direction.

**Figure 5:**
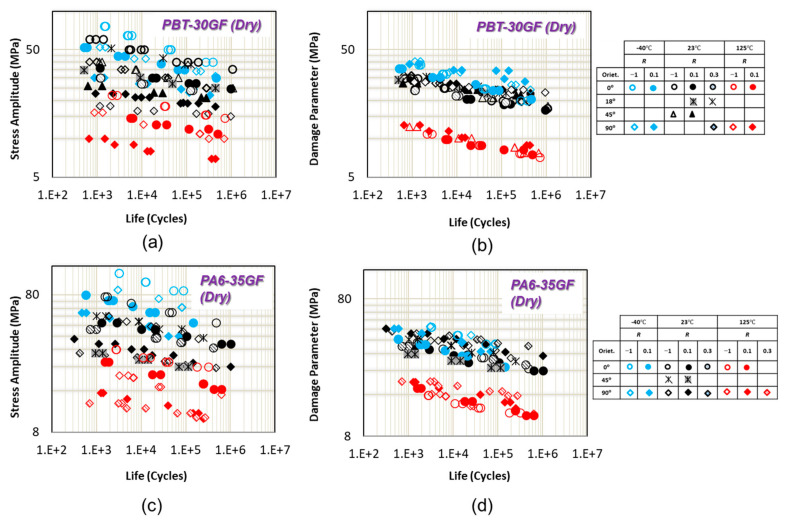
(**a**) Uniaxial fatigue data of PBT-30GF from [6]. (**b**) Uniaxial fatigue data of PA6-35GF from [6]. (**c**) Uniaxial fatigue data correlations of PBT-30GF using the proposed damage parameter. (**d**) Uniaxial fatigue data correlations of PA6-35GF using the proposed damage parameter.

The uniaxial fatigue data for PBT-GF30 (dry) from [6] and PA6-GF35 (dry) from [6] with four fiber orientations are shown in Figure 5a,c, respectively, at three different *R* ratios. The loading conditions are listed in Table 1. As can be seen from these figures, fatigue performance is highly dependent on the fiber orientation. The effect of fiber orientation on PBT-GF30 (dry) and PA6-GF35 (dry) at both stress ratios linearly increases with increasing temperature [6]. For PBT-GF30 (dry) with fibers along loading direction, crack propagation is blocked by fibers (fiber avoiding mechanism) and crack grows along the fiber interface. On the other hand, in specimens with 90° fiber orientation, a nearly flat fracture surface along the fiber–matrix interface was observed [6].

As can be seen in Figure 5b,d, all uniaxial fatigue data at different *R* ratios and fiber orientations for both materials and at all tested temperatures of −40 °C, 23 °C, and 120 °C correlate together using damage parameter (Equation (3)). Fiber orientation effect is accounted for through the third term in parenthesis in Equation (3). However, the correlations are better for PBT-30GF, as compared to PA6-35GF.

**Figure 6:**
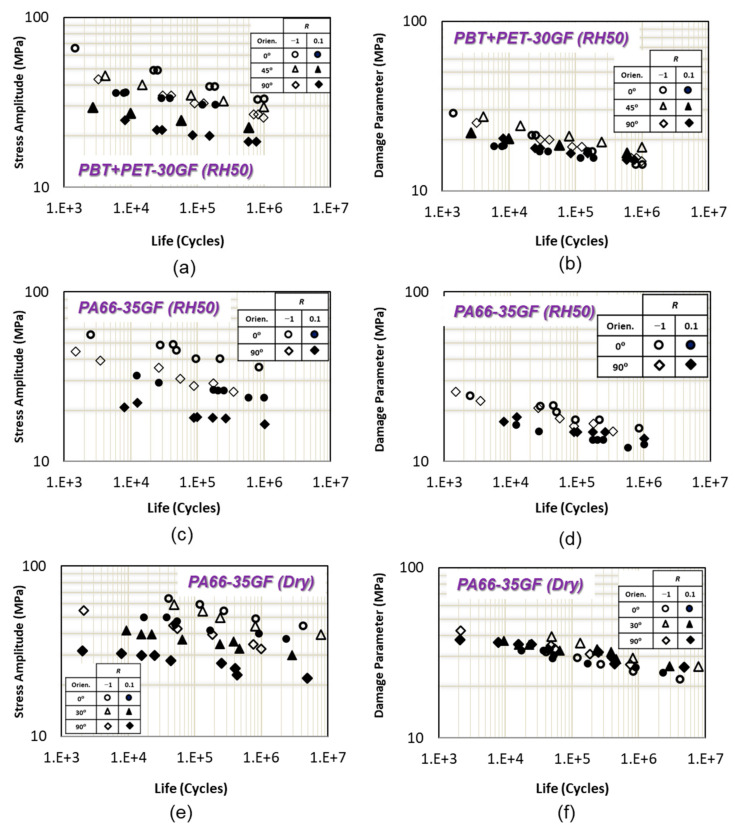
(**a**) Uniaxial fatigue data of PBT + PET-30GF from [9]. (**b**) Uniaxial fatigue data correlations of PBT + PET-30GF using the proposed damage parameter. (**c**) Uniaxial fatigue data of PA66-35GF with 50% relative humidity from [17]. (**d**) Uniaxial fatigue data correlations of PA66-35GF with 50% relative humidity with the proposed damage parameter. (**e**) Uniaxial fatigue data of PA66-35GF with dry condition from [18]. (**f**) Uniaxial fatigue data correlations of PA66-35GF with dry condition with the proposed damage parameter.

Mean stress and fiber orientation effects on uniaxial fatigue life of PBT+PET-GF30 (RH50), PA66-GF35 (RH50), and PA66-GF35 (dry) at 23 °C are shown in Figure 6a,c,e. Fiber orientation has a significant effect on fatigue life for PBT + PET-GF30 and PA66-GF35 (RH50) at the same *R* ratio. Flat fracture surface and zigzag crack growth pattern were observed for samples with 90° and 0° fiber orientations, respectively [9]. Therefore, matrix/fiber interface is a critical location for crack initiation. Fiber orientation affects fatigue performance of PA66-GF35 (dry) specimens with 90° fiber orientation much more than specimens with 0° or 30° fiber orientation, as can be seen in Figure 6c.

Tensile mean stress has a significant detrimental effect on fatigue life of PBT + PET-GF30 (RH50) and PA66-GF35 (dry) when fiber orientation increases from 0° to 90°. However, the reducing effect of tensile mean stress on fatigue life is the same for PA66-GF35 (RH50) both fiber orientations as shown in Figure 6c.

Uniaxial fatigue data at different *R* ratios and for different fiber orientations correlate well using the proposed damage parameter for PBT + PET-GF30, PA66-GF35 (RH50), and PA66-GF35 (dry), as shown in Figure 6b–f, respectively. The correlation of data in this section indicate that the damage parameter can account for mean stress and fiber orientation effects well.

## 6. Incorporation of the Proposed Damage Parameter in a General Fatigue Model

A general fatigue life prediction model based on the strength degradation concept under constant amplitude loading was suggested and applied to the experimental uniaxial fatigue data of continuous glass fiber-reinforced plastic composites in [24]. A simplified version of this model can be expressed as:

(6) $$S_{u}-S_{max}=-\alpha S_{u}^{1-\xi }\;S_{max}^{\xi }\;{\left(1-R\right)}^{\xi }(N_{f}^{\beta }-1),$$

where *α* and *β* are material constants, $S_{max}$ is maximum stress, and *ξ* is an empirical function of stress ratio, *R*, and mold flow direction, *θ*, expressed as [24]:

(7) ξ=1.6−Rsinθ,

This model was later used for a variety of neat and SGFR thermoplastics for uniaxial fatigue life prediction under different loading conditions including cyclic frequency and mean stress as well as effects of temperature and humidity through ultimate tensile strength at the relevant temperature and humidity [6,25] with a form expressed as:

(8) $$S_{u}-S_{max}=-\alpha S_{u}^{1-\xi }\;S_{max}^{\xi }\;{\left(1-R\right)}^{\xi }\frac{1}{f^{\beta }}(N_{f}^{\beta }-1),$$

where f is the cycling frequency. The equivalent stress based on this equation can be expressed as [25]:

(9) $$S_{eq}=A^{\prime }{\left\{\frac{f^{\beta }\left[S_{u}-\frac{\Delta S}{1-R}\right]}{\alpha S_{u}^{1-\xi }{\left(\Delta S\right)}^{\xi }}+1\;\right\}}^{\frac{b}{\beta }}=A^{\prime }{\left(N_{f}\right)}^{b},$$

where *A*′ = 96.3 MPa and *b* = −0.072 are the axial fatigue intercept and exponent in the reference condition, here considered as fully reversed (*R* = −1) fatigue data of PBT-35GF in the longitudinal direction at 23 °C. The correlations of the fatigue life predictions were shown to be very good for the uniaxial condition where *β* was taken equal to 0.2 for all materials, as was also the case in [25].

**Figure 7:**
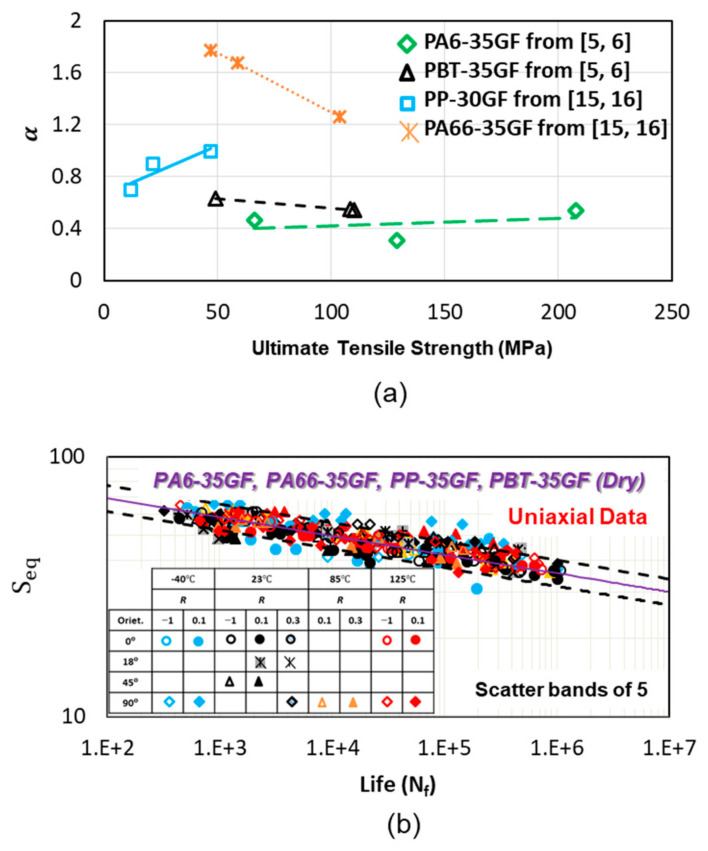
(**a**) Constant *α* used in general model as a function of ultimate tensile strength. (**b**) Uniaxial fatigue data correlations of PA66-30GF, PP-30GF, PBT-35GF, PA6-35GF using the proposed damage parameter and the general model from [5,6,15,16].

The critical plane damage parameter can be incorporated in the general model as well. Equation (3) was used to calculate the damage parameter for SGFR thermoplastics. Therefore, by using *R* = −1 and $S_{a}=D_{C}$ in Equation (9) the final form of general fatigue model for uniaxial loading can be expressed as:

(10) $$S_{eq}=A^{\prime }{\left\{\frac{f^{\beta }\left[S_{u}-D_{C}\right]}{\alpha S_{u}^{1-\xi }{\left(2D_{C}\right)}^{\xi }}+1\;\right\}}^{\frac{b}{\beta }},$$

where *α* is a material and temperature and humidity dependent constant where it can be estimated based on the ultimate tensile strength of the material as shown in Figure 7a. *α* can be estimated as a linear function of ultimate tensile strength:

(11) $$\alpha =A\;S_{u}+B,$$

where *A* and *B* are the empirical constants and reported in Table 3 for each material. In order to calculate the constant *α* for each material, reference condition was defined as longitudinal direction (transverse direction was used if longitudinal direction data were not available) under fully reversed axial loading at each temperature. Values of α are different for PP, PBT, PA6, and PA66 from what had been used in [6,25] due to using *R* = −1 and $S_{a}=D_{C}$ in Equation (9).

It should be noted that the *R* value in Equation (7) for *ξ* is taken as *R* = −1 since the mean stress or *R* ratio correction is already accounted for in the damage model, $D_{C}$. Similarly, the effect of fiber orientation is already accounted for in the proposed damage model. Therefore, either *θ* = 0 or *θ* = 90° was used in computing *ξ* from Equation (7), depending on the fiber orientation with respect to the loading direction for the baseline data set of a given material. These considerations result in the value of being either 1.6 or 2.6 in Equation (10).

The uniaxial fatigue data correlations using the proposed critical plane damage parameter and general fatigue model for PA66-30GF, PP-30GF, PBT-35GF, and PA6-GF are shown in Figure 7b. The test frequency range for all materials was 0.25–10 Hz, depending on the stress level and temperature. The critical plane damage parameter accounts for fiber orientation and mean stress effects. Calculated damage parameter $D_{c}$, at different temperatures and frequencies were used as the input for the general fatigue model. Therefore, altogether fiber orientation (0°, 18°, 45°, and 90°), mean stress (*R* = −1, *R* = 0.1, and *R* = 0.3), temperature (−40 °C, 23 °C, 85 °C, and 125 °C), and loading rate effects (0.25–10 Hz) are considered for all the materials and reasonably located within relatively narrow scatter bands, as shown in the Figure 7b.

## 7. Conclusions

Uniaxial fatigue behavior of injection-molded short glass fiber-reinforced thermoplastics were studied under different environmental and loading conditions. A critical plane damage model is presented in this study for uniaxial life predictions of reinforced short glass fiber thermoplastics based on the observed damage mechanisms of short glass fiber polymer composites. Uniaxial fatigue data for a variety of SGFR thermoplastics under different loading and environmental conditions from the literature were used in the analysis and modeling. Based on the analysis results, the following conclusions can be made:

1. The proposed critical plane damage model successfully accounts for fiber orientation and mean stress effects under uniaxial loading condition of considered materials at different temperatures. Effect of mean stress is accounted for by using maximum normal stress. Effect of fiber orientation is considered through the projections of each normal and shear stresses along the fiber;
2. The data correlations for PA66-35GF under dry and RH50 using a modified energy criterion were located within scatter bands more than 5 [26] while the data correlation using damage parameter is within scatter bands of 5. The data correlation using damage parameter is somewhat better than the energy parameter used in [26]. In addition, unlike the energy parameter the damage parameter is consistent with the failure mechanisms in the reinforced composite;
3. Using the damage parameter combined with a general fatigue model, it was possible to also include the effects of cycling frequency and temperature. The constant for the general model can be estimated from the ultimate tensile strength of the material at different temperatures or relative humidity;
4. The application of the proposed critical plane damage model can be extended to multiaxial loading condition since it successfully correlated fatigue data for short fiber-reinforced thermoplastics with different fiber orientations under uniaxial loading condition.

## Figures and Tables

**Table 1:** Literature data sets for the mechanical and physical properties of injection-molded short glass fiber composites used in this study. Fiber diameter is 10 μm. The fiber aspect ratio (fiber length/fiber diameter) range is 23–26.

Ref.	Matrix	Reinf.	Fiber Length (μm)	*T_g_* (°C)	*T_m_* (°C)	T (°C)	Fiber Orin.	*E* (Gpa)	*S_u_* (MPa)
[5,6]	PBT, Dry	35% GF	253	60	220	−40	0°	9.7	140
						−40	90°	6.4	74
						23	0°	8.6	109
						23	18°		
						23	45°	5.4	69
						23	90°	5.8	61
						125	0°	3.3	49
						125	90°	1.8	27
[5,6]	PA6 + Rubber, Dry	35% GF	253	60	220	−40	0°	10	207
						−40	90°	5.9	132
						23	0°	7.2	130
						23	45°	4.9	87
						23	90°	4.3	87
						125	0°	1.2	66
						125	90°	2.9	37
[15,16]	PP, Dry	30% GF	250	0	165	23	90°	3.4	46.8
						85		1.5	21.8
						120		0.95	11.9
[15,16]	PA66, Dry	30% GF	250	50	260	23	90°	5.2	103.5
						85		2.25	59.0
						120		1.6	46.8
[9]	PBT-PET, 50% RH	30% GF	230	50		23	0°		
							45°		
							90°		
[17]	PA66, 50% RH	30% GF	230	25		23	0°		
							90°		
[18]	PA66, Dry	30% GF	240–280	65	285	23	0°	10,000	160
							30°		
							90°		

**Table 2:** Literature data sets for un-notched uniaxial fatigue behavior of injection-molded short glass fiber composites. Fiber diameter is 10 μm. The fiber aspect ratio (fiber length/fiber diameter) range is 23–26.

Ref.	Thi (mm)	Matrix	Reinf.	RH ^1^ (%)	Fiber Orin.	T (°C)	*R_σ_*
[5,6]	3.8	PBT	35% GF	dry	0°	−40	−1, 0.1
					0°	23	−1, 0.1
					0°	125	−1, 0.1
					18°	23	−1, 0.1
					45°	23	−1, 0.1
					90°	−40	−1, 0.1
					90°	23	−1, 0.1
					90°	125	−1, 0.1
[5,6]	3.8	PA6 + Rubber	35% GF	dry	0°	−40	−1, 0.1
					0°	23	−1, 0.1
					0°	125	−1, 0.1
					45°	23	−1, 0.1
					90°	−40	−1, 0.1
					90°	23	−1, 0.1
					90°	125	−1, 0.1, 0.3
[15,16]	2.8	PP	30% GF	dry	90°	23	0.1, 0.3
						85	0.1, 0.3
						120	0.1, 0.3
[15,16]	2.8	PA66	30% GF	dry	90°	23	0.1, 0.3
						85	0.1, 0.3
						120	0.1, 0.3
[9]	3	PBT-PET	30% GF	50	0°	23	−1, 0
					45°	23	−1, 0
					90°	23	−1, 0
[17]	3	PA66	30% GF	50	0°	23	−1, 0
					90°	23	−1, 0
[18]	1	PA66	30% GF	dry	0°	23	−1, 0
					30°	23	−1, 0
					90°	23	−1, 0

^1^ Relative Humidity.

**Table 3:** FS and general model constants for considered materials under different environmental conditions.

Ref.	Material	T (°C)	RH ^1^ (%)	*k*	*α*	*A*	*B*
[15,16]	PA66-30GF	23	Dry	0.2	1.257	−0.0091	2.204
		85		0.2	1.674		
		120		0.2	1.77		
[15,16]	PP-30GF	23	Dry	0.2	0.95	0.0058	0.708
		85		0.2	0.94		
		120		0.2	0.7		
[5,6]	PBT-35GF	−40	Dry	0.2	0.54	−0.0014	0.6974
		23		0.2	0.55		
		125		0.2	0.63		
[5,6]	PA6-35GF	−40	Dry	0.1	0.54	0.0006	0.3605
		23		0.2	0.31		
		125		0.2	0.47		

^1^ Relative Humidity.

## Data Availability

The data presented in this study are available upon request from the corresponding author.
